# Induction of broad multifunctional CD8+ and CD4+ T cells by hepatitis B virus antigen-based synthetic long peptides *ex vivo*

**DOI:** 10.3389/fimmu.2023.1163118

**Published:** 2023-09-13

**Authors:** Diahann T. S. L. Jansen, Monique T. A. de Beijer, Robbie J. Luijten, Kitty Kwappenberg, Anna-Sophia Wiekmeijer, Amy L. Kessler, Roel F. A. Pieterman, Rachid Bouzid, Willem-Jan Krebber, Robert A. de Man, Cornelis J. M. Melief, Sonja I. Buschow

**Affiliations:** ^1^Department of Gastroenterology and Hepatology, Erasmus MC University Medical Center Rotterdam, Rotterdam, Netherlands; ^2^ISA Pharmaceuticals B.V., Oegstgeest, Netherlands

**Keywords:** HBV, HLA, T cell, chronic hepatitis B, therapeutic vaccination, synthetic long peptide

## Abstract

**Introduction:**

Therapeutic vaccination based on synthetic long peptides (SLP^®^) containing both CD4+ and CD8+ T cell epitopes is a promising treatment strategy for chronic hepatitis B infection (cHBV).

**Methods:**

We designed SLPs for three HBV proteins, HBcAg and the non-secreted proteins polymerase and X, and investigated their ability to induce T cell responses *ex vivo*. A set of 17 SLPs was constructed based on viral protein conservation, functionality, predicted and validated binders for prevalent human leukocyte antigen (HLA) supertypes, validated HLA I epitopes, and chemical producibility.

**Results:**

All 17 SLPs were capable of inducing interferon gamma (IFNɣ) production in samples from four or more donors that had resolved an HBV infection in the past (resolver). Further analysis of the best performing SLPs demonstrated activation of both CD8+ and CD4+ multi-functional T cells in one or more resolver and patient sample(s). When investigating which SLP could activate HBV-specific T cells, the responses could be traced back to different peptides for each patient or resolver.

**Discussion:**

This indicates that a large population of subjects with different HLA types can be covered by selecting a suitable mix of SLPs for therapeutic vaccine design. In conclusion, we designed a set of SLPs capable of inducing multifunctional CD8+ and CD4+ T cells *ex vivo* that create important components for a novel therapeutic vaccine to cure cHBV.

## Introduction

Chronic infection with the Hepatitis B virus (HBV) is a major global health burden that currently affects approximately 240 million people worldwide (1). Treatment with nucleos(t)ide analogs (NAs) can suppress viral replication but offers neither cure nor full abolition of the chronic HBV infection (cHBV)-related risk for hepatocellular carcinoma (HCC) or liver cirrhosis. Importantly, NAs need to be taken for life (2).

At present, a functional cure, in which the viral surface antigen (HBsAg) is cleared from the circulation with or without concomitant HBsAg-directed antibodies, is considered the desired therapy outcome (3). To achieve this, not only complete suppression of viral replication and protein production but also empowerment of the HBV-directed T cell response is likely needed (4). As reviewed recently by us and others, one way to achieve this is via therapeutic vaccination (5, 6). As outlined in these reviews, several attempts towards therapeutic vaccines for HBV clearance have been made yet have shown no clear clinical effect. Immunological effects were observed in some studies but were often not investigated in great detail and hence offered no insight into why these vaccines were ineffective. Furthermore, the design of past vaccines may have been suboptimal. Very often the vaccine targeted only one viral protein or peptide (mostly HBsAg) or were not optimal for the induction of T cell responses because of the applied adjuvant or vaccine platform (6). To achieve a long-lasting CD8+ T cell response able to clear an existing viral infection, the activation of both anti-viral CD8+ T cell responses as well as CD4+ T helper responses against multiple viral targets are needed (7–9).

In the last two decades, synthetic long peptides (SLP) have surfaced as a highly effective platform to induce both CD4+ and CD8+ T cell responses (10). SLPs are typically 20-40 amino acids long and because of this length are more efficiently processed for presentation on human leukocyte antigens (HLA) than whole proteins (11, 12). Each SLP can contain multiple epitopes for a variety of HLA types. Moreover, multiple SLPs (i.e., 10-20 SLPs) can be used in a single vaccine, further increasing epitope coverage. Furthermore, SLP vaccines are well tolerated and have demonstrated efficacy in chronic infection with human papilloma virus (HPV) and multiple cancers, either as stand-alone therapy or in combination with other forms of immunotherapy like PD-1 blockade (13, 14). SLPs can also easily be combined or even conjugated to different vaccine adjuvants (15). Previously, we have demonstrated the ability of a prototype SLP based on the HBV core protein (HBcAg) containing the immunodominant HBcAg18-27 epitope to activate CD8+ T cell responses against this epitope using cHBV patient blood cells *ex vivo* (16).

Here, we expand this work to other HBV proteins, also including the viral polymerase (Pol) and X proteins. T cells specific for HBV proteins HBcAg, Pol, and X are still detected in the blood of many cHBV patients and may have suffered less from antigen-driven immune exhaustion than HBsAg specific T cells (17–19). T cell responses against both HBcAg and Pol have been associated with viral immune control in cHBV (20, 21). Responses to X are in general less numerous, yet the X protein is essential for viral protein translation and maintenance of viral replication. T cell responses to X have been observed in patients clearing the infection (22–25).

We set out to develop an off-the-shelf therapeutic SLP vaccine based on these three proteins that, especially when combined into a single vaccine, can be used for all cHBV patients regardless of their HLA type and HBV genotype. We focused on the most conserved parts of these proteins with demonstrated functional relevance and a high epitope density (26). We developed 17 SLPs that were then extensively tested *in vitro* using blood of both HBV resolvers and cHBV patients. We demonstrate that single SLPs can activate both CD4+ and CD8+ T cells responses, but for a different set of individuals and based on different epitopes. Pools of five SLPs, however, provided a good, almost complete, coverage in both patients and resolvers. These data illustrate the direction of HBV SLP design toward therapeutic HBV vaccine development.

## Materials and methods

### HBV resolvers and chronic HBV patients

Peripheral blood mononuclear cells (PBMCs) were isolated from buffy coats by Ficoll (GE Healthcare) density centrifugation from 17 blood donors, who had previously resolved an acute hepatitis B infection shown by the presence of anti-HBcAg antibodies (resolver; rHBV), and three healthy donors with no anti-HBV antibodies. Buffy coats were provided by the local blood bank including two-digit HLA typing and informed consent (Sanquin). To obtain PBMCs from chronic HBV patients, blood was collected from five patients with low serum viral load (HBV DNA ≤ 1000 IU/mL), serum ALT levels below the upper limit of normal (56 IU/L), and low to moderate fibrosis (fibroscan F0-F2). All patients were HBeAg negative and 80% received nucleos(t)ide analogs. All patients were negative for antibodies against hepatitis C, hepatitis D, and human immunodeficiency virus. See **Table 1** for all donor characteristics.

**Table 1:** Patient and donor characteristics.

Donor #	Gender	Age	Viral load (IU/mL)	ALT (IU/L)	HBeAg	Fibrosis	Genotype	Ethnicity	Current Therapy	Comment
Donor 1	M	65	n/a	n/a	n/a	n/a	n/a	n/a	n/a	Resolver
Donor 2	F	58	n/a	n/a	n/a	n/a	n/a	n/a	n/a	Resolver
Donor 3	M	50	n/a	n/a	n/a	n/a	n/a	n/a	n/a	rHBV6
Donor 4	F	62	n/a	n/a	n/a	n/a	n/a	n/a	n/a	rHBV1
Donor 5	M	58	n/a	n/a	n/a	n/a	n/a	n/a	n/a	Resolver
Donor 6	F	70	n/a	n/a	n/a	n/a	n/a	n/a	n/a	rHBV5
Donor 7	F	51	n/a	n/a	n/a	n/a	n/a	n/a	n/a	Resolver
Donor 8	M	67	n/a	n/a	n/a	n/a	n/a	n/a	n/a	Resolver
Donor 9	M	67	n/a	n/a	n/a	n/a	n/a	n/a	n/a	Resolver
Donor 10	M	62	n/a	n/a	n/a	n/a	n/a	n/a	n/a	Resolver
Donor 11	F	55	n/a	n/a	n/a	n/a	n/a	n/a	n/a	Resolver
Donor 12	M	64	n/a	n/a	n/a	n/a	n/a	n/a	n/a	Resolver
Donor 13	M	52	n/a	n/a	n/a	n/a	n/a	n/a	n/a	Resolver
Donor 14	F	54	n/a	n/a	n/a	n/a	n/a	n/a	n/a	rHBV4
Donor 15	F	24	n/a	n/a	n/a	n/a	n/a	n/a	n/a	Resolver
Donor 16	F	35	n/a	n/a	n/a	n/a	n/a	n/a	n/a	Healthy
Donor 17	M	55	n/a	n/a	n/a	n/a	n/a	n/a	n/a	Healthy
Donor 18	F	65	n/a	n/a	n/a	n/a	n/a	n/a	n/a	Healthy
	F	65	n/a	n/a	n/a	n/a	n/a	n/a	n/a	rHBV2
	F	60	n/a	n/a	n/a	n/a	n/a	n/a	n/a	rHBV3
cHBV1	M	36	7.01E2	51	neg	F2	D	Other	None	
cHBV2	M	64	< 2.00E1	34	neg	F0-F1	B	Asian	Entecavir	
cHBV3	M	28	3.20E1	55	neg	F0-F1	A	Caucasian	Tenofovir	
cHBV4	M	39	< 2.00E1	23	neg	F0-F1	n.d.	Other	Entecavir	
cHBV5	F	46	< 2.00E1	20	neg	F0-F1	C	Asian	Tenofovir	

M, male; F, female; n/a, not applicable; ALT, alanine aminotransferase; n.d., not determined.

Other; ethnicities not covered by Caucasian, Asian, or African.

### Peptides

**Figure 1:**
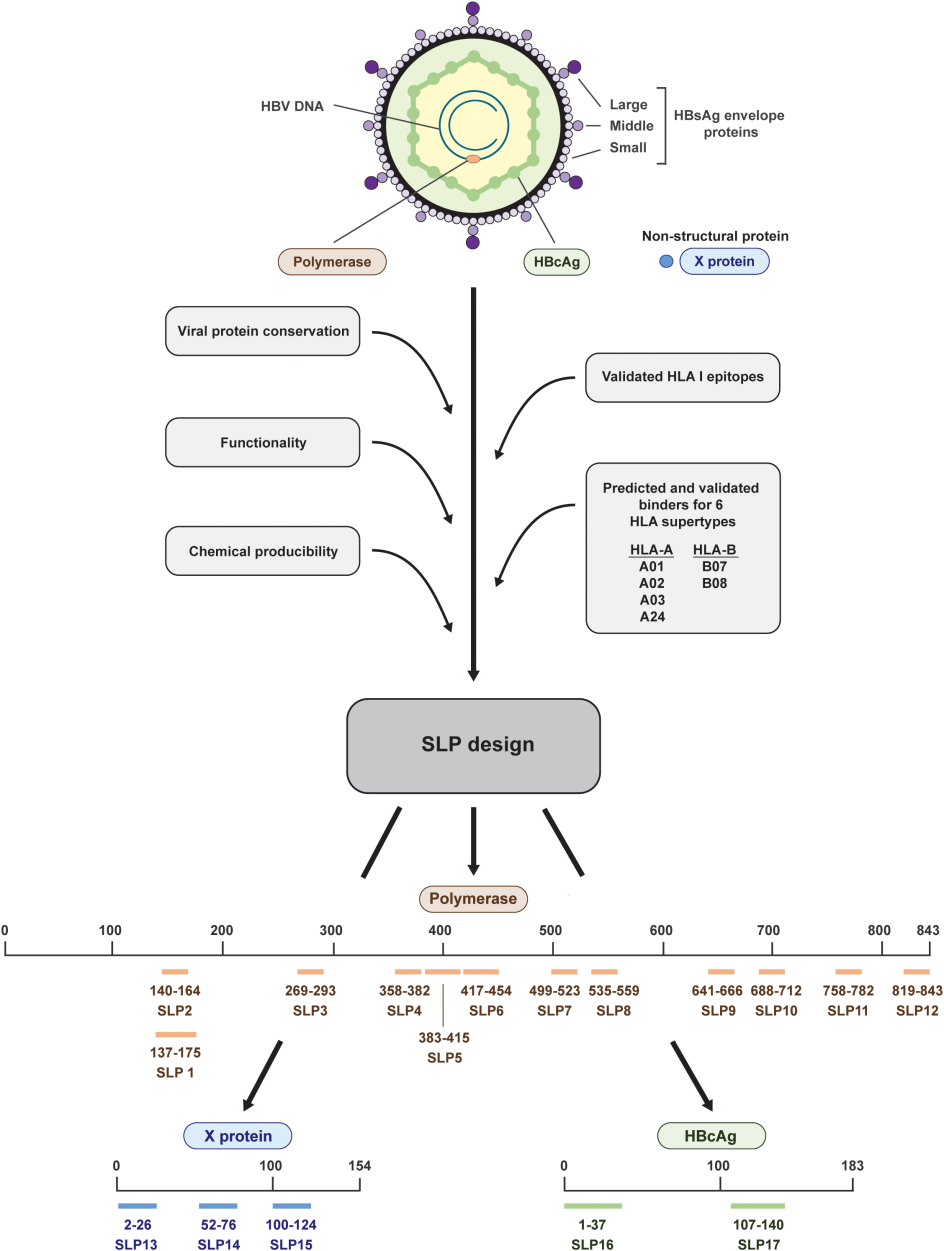
Design and placement in the HBV proteins of the 17 synthetic long peptides. The HBV proteins polymerase (salmon), HBcAg (green), and X (blue) were selected for the design of synthetic long peptides (SLPs). Based on the conserved parts of the proteins, functional domains, validated HLA I epitopes, predicted and validated binders for six HLA supertypes, and chemical producibility, 17 SLPs were designed. In total, 12 SLPs originated from polymerase (salmon; SLP1-12), 3 SLPs originated from the X protein (blue; SLP13-15), and 2 SLPs originated from the HBcAg protein (green; SLP16 and 17). The numbering indicates the position within the proteins.

All SLPs were produced by ISA Pharmaceuticals BV, Leiden, the Netherlands, according to the design described in the results section (**Figure 1**, **Table 2**). They were generated using solid phase Fmoc/tBu chemistry on an Advanced ChemTech TETRAS peptide synthesizer and purified to at least 95% on a Gilson preparative HPLC system. The identity and purity of the peptides was confirmed with ultra-performance liquid chromatography-mass spectrometry (UPLC-MS) on a Waters ACQUITY UPLC/TQD system. SLPs were dissolved in 10% dimethyl sulfoxide (DMSO), 90% H_2_O, and stored at −20°C.

**Table 2:** Overview of synthetic long peptides.

SLP#	PROTEIN	POSITION	SEQUENCE
**SLP1**	Polymerase	137-175	VVNHYFQTRHYLHTLWKAGILYKRETTRSASFCGSPYSW
**SLP2**	Polymerase	140-164	HYFQTRHYLHTLWKAGILYKRETTR
**SLP3**	Polymerase	269-293	ASSSSSCLHQSAVRKAAYSHLSTSK
**SLP4**	Polymerase	358-382	HHIRIPRTPARVTGGVFLVDKNPHN
**SLP5**	Polymerase	383-415	TAESRLVVDFSQFSRGITRVSWPKFAVPNLQSL
**SLP6**	Polymerase	417-454	NLLSSNLSWLSLDVSAAFYHIPLHPAAMPHLLIGSSGL
**SLP7**	Polymerase	499-523	RKLHLYSHPIILGFRKIPMGVGLSP
**SLP8**	Polymerase	535-559	SVVRRAFPHCLAFSYMDDVVLGAKS
**SLP9**	Polymerase	641-666	GFAAPFTQCGYPALMPLYACIQAKQA
**SLP10**	Polymerase	688-712	ARQRPGLCQVFADATPTGWGLAIGH
**SLP11**	Polymerase	758-782	TSFPWLLGCAANWILRGTSFVYVPS
**SLP12**	Polymerase	819-843	SPSVPSHLPDRVHFASPLHVAWRPP
**SLP13**	X protein	2-26	AARLCCQLDPARDVLCLRPVGAESR
**SLP14**	X protein	52-76	HLSLRGLPVCAFSSAGPCALRFTSA
**SLP15**	X protein	100-124	LSAMSTTDLEAYFKDCLFKDWEELG
**SLP16**	HBcAg	1-37	MDIDPYKEFGATVELLSFLPSDFFPSVRDLLDTASAL
**SLP17**	HBcAg	136-169	CLTFGRETVLEYLVSFGVWIRTPPAYRPPNAPIL

Peptides (9-10mers and 15-16mers) selected for further analysis derived from SLP7 (pol499-523), SLP14 (x52-76), and SLP16 (c1-37) were purchased from Peptide 2.0.

### IFNγ ELISpot

Cryopreserved PBMCs were thawed and rested for 45-60 minutes at 37°C before transfer to an anti-IFNγ-coated (1-D1K, 5 μg/mL, Mabtech) 96-well polyvinylidene fluoride (PVDF) plate (Millipore). PBMCs were seeded at 200,000 cells per well in quadruplicates and stimulated with 10 μM of the indicated SLP for 22 hours at 37°C. The CEF peptide pool (2 μg/mL, Mabtech), consisting of 23 HLA I-restricted peptides derived from human cytomegalovirus (CMV), the Epstein–Barr virus (EBV), and influenza virus, and phytohemagglutinin (PHA) (1 μg/mL, Sigma) were taken along as positive controls in addition to a peptide solvent (10% DMSO, 90% H_2_O) condition as negative control. The plate was developed using anti-IFNγ-biotin (7-B6-1, 0.3 μg/mL, Mabtech), Streptavidin-ALP (1 μg/mL, Mabtech), and BCIP/NBT-plus substrate (100 μL/well, Mabtech). The color process was stopped with water and spots were counted using an S6 Ultimate Immunospot Analyzer (Cellular Technology Limited LLC). Specific spots were calculated using two different methods: 1) the average number of spots in four wells subtracted by the average number of spots in the four corresponding DMSO control wells + 2x standard deviation; and 2) the cumulative number of spots in four wells subtracted by the cumulative number of spots in the four DMSO control wells.

### Expansion and activation of SLP-specific T cells

Frozen PBMCs (5x10^6^-10x10^6^) were cultured in T25 culture flasks in Iscove’s modified Dulbecco’s medium (IMDM) (Lonza) + 2% human serum (Sanquin) + 1% penicillin-streptomycin (Gibco) + 1% Ultraglutamine (Gibco) at 1x10^6^ cells/mL and a mix of five SLPs, 3 μM each, to induce expansion of SLP-specific T cells. For each rHBV (n=6) or cHBV (n=5), SLPs were distributed over two pools and immediately tested; SLP Mix 1 contained SLP5, SLP7, SLP13, SLP15, and SLP17; SLP Mix 2 contained SLP6, SLP8, SLP10, SLP14, and SLP16. On day 3, 50 IU/mL IL-2 (Miltenyi) was added to facilitate the expansion of the SLP-specific T cells and fresh medium containing interleukin-2 (IL-2) was added again on day 5, 7, and 10. On day 12, cells only received fresh medium (without IL-2) to let the cells rest before they were restimulated with the SLP mix or the single SLPs on day 13-14. After 22 hours of restimulation in 96-well round bottom plates in quadruplicate, supernatant was collected to analyze the produced cytokines (IFNγ, tumor necrosis factor (TNF)α, TNFβ, GM-CSF, IL-2, IL-3, Il-4, IL-5, IL-6, IL-8, IL-9 IL-10, IL-13, and IL-22) in response to stimulation via multiplex cytokine analysis (Procartaplex, ThermorFisher) measured on a MAGPIX (Luminex). The concentrations of IFNγ and TNFα were calculated by subtraction of the average negative control value plus two times the standard deviation. Restimulations with peptide solvent control DMSO in the concentration representative of a single SLP/SLP-mix and irrelevant PRAME peptide (T cell epitope in melanoma) were used as negative controls. The cells were used to determine the expression of activation markers CD69, CD134, and CD137 on CD4+ and CD8+ T cells using flowcytometry after restimulation. The difference between the positive percentage in the two negative DMSO controls (representative of single-SLP and mixed-SLP DMSO concentrations) was used to correct for background as only two instead of three negative controls were used. This difference (instead of standard deviation) was multiplied by two and added to the percentage measured in the DMSO control sample and this value was subtracted from the positive percentages in the different samples.

To analyze the activation of SLP-specific T cells after stimulation with SLP-contained peptides, cryopreserved PBMCs from selected donors expanded with the SLP pools as described above were stimulated with the SLP or the corresponding peptides in the presence of 1 μg/mL anti-CD28 and 1 μg/mL anti-CD49d antibodies (both Biolegend) for a total of 6 hours. Brefeldin A (10 μg/mL, Sigma) was added for the last 5 hours and the production of IFNγ and TNFα was determined by intracellular cytokine staining. Cytokine response was determined by subtraction of the average DMSO value of three DMSO control samples per donor plus two times the standard deviation.

### Flow cytometry

For phenotyping of the total T cell populations, PBMCs were thawed, rested for 4 hours at 37°C, and stained with previously described panels for T cell maturation markers and T cell co-inhibitory receptors (27). Positive populations were determined using fluorescence minus one (FMO) controls and measurements were corrected for background fluorescence (**Supplementary Figure 1**).

For the analysis of T cell activation after 14 days of SLP-induced expansion and restimulation, cells were stained for 30 minutes at 4°C in the dark with an activation marker panel including the following antibodies; CD3 (SK7, eBioscience), CD4 (SK3, BD Biosciences), CD8 (RPA-T8, eBioscience), CD69 (FN50, Biolegend), CD137 (4B4-1, Biolegend), CD134 (ACT-35, Biolegend), CD14 (61D3, eBioscience), CD19 (HIB19, eBioscience), CD56 (NCAM16, BD Biosciences), and LIVE/DEAD Fixable Green (Invitrogen). Isotype control stainings were performed for CD137, CD134, and CD69 (**Supplementary Figure 2**). Samples were measured on a BD FACSCanto II (BD Biosciences).

For the assessment of cytokine production, stimulated cells were first stained with a mix of antibodies staining surface markers; CD3 (SK7, eBioscience), CD4 (SK3, BD Biosciences), CD8 (RPA-T8, eBioscience), CD56 (MY31, BD Biosciences), CD14 (MOP9, BD Biosciences), CD16 (ebioCB16, eBioscience), and LIVE/DEAD Fixable Aqua (Invritogen), followed by fixation with 2% formaldehyde and permeabilization with 0.5% saponin. Cells were next stained with IFNγ (25723.11, BD Biosciences) and TNFα (Mab11, eBioscience) or isotype control antibodies and acquired on a BD FACSCanto II (BD Biosciences) (**Supplementary Figure 3**). All flowcytometry data were analyzed using FlowJo version 10 software (Tree Star).

### Statistical analysis

Unpaired non-parametric (Mann–Whitney) tests were performed using the GraphPad Prism 8 software (GraphPad Software). P-values of <0.05 were considered statistically significant.

## Results

### Synthetic long peptides based on HBV proteins all elicit an IFNɣ response in PBMCs of several HBV resolvers

Taking into account chemical manufacturability, SLPs were constructed to maximally cover predicted and validated binders (CD8+ T cell epitopes) for six HLA I supertypes that together cover around 95% of the world population and to include conserved and functional protein domains (26, 28–30) (**Figure 1**). Less focus was put on HLA II (CD4+ T cell) epitopes as these are more difficult to predict and HLA II binding is more promiscuous than HLA I binding (31–33). Because of the latter, however, one or more HLA II epitopes are likely already included in each SLP by chance. In total, we designed and synthetized 17 SLPs; 12 SLPs originating from the very large polymerase protein (SLP1-12), 3 SLPs originating from the X protein (SLP13-15), and 2 SLPs originating from the HBcAg protein (SLP16 and 17) (**Figure 1**, **Table 2**).

**Figure 2:**
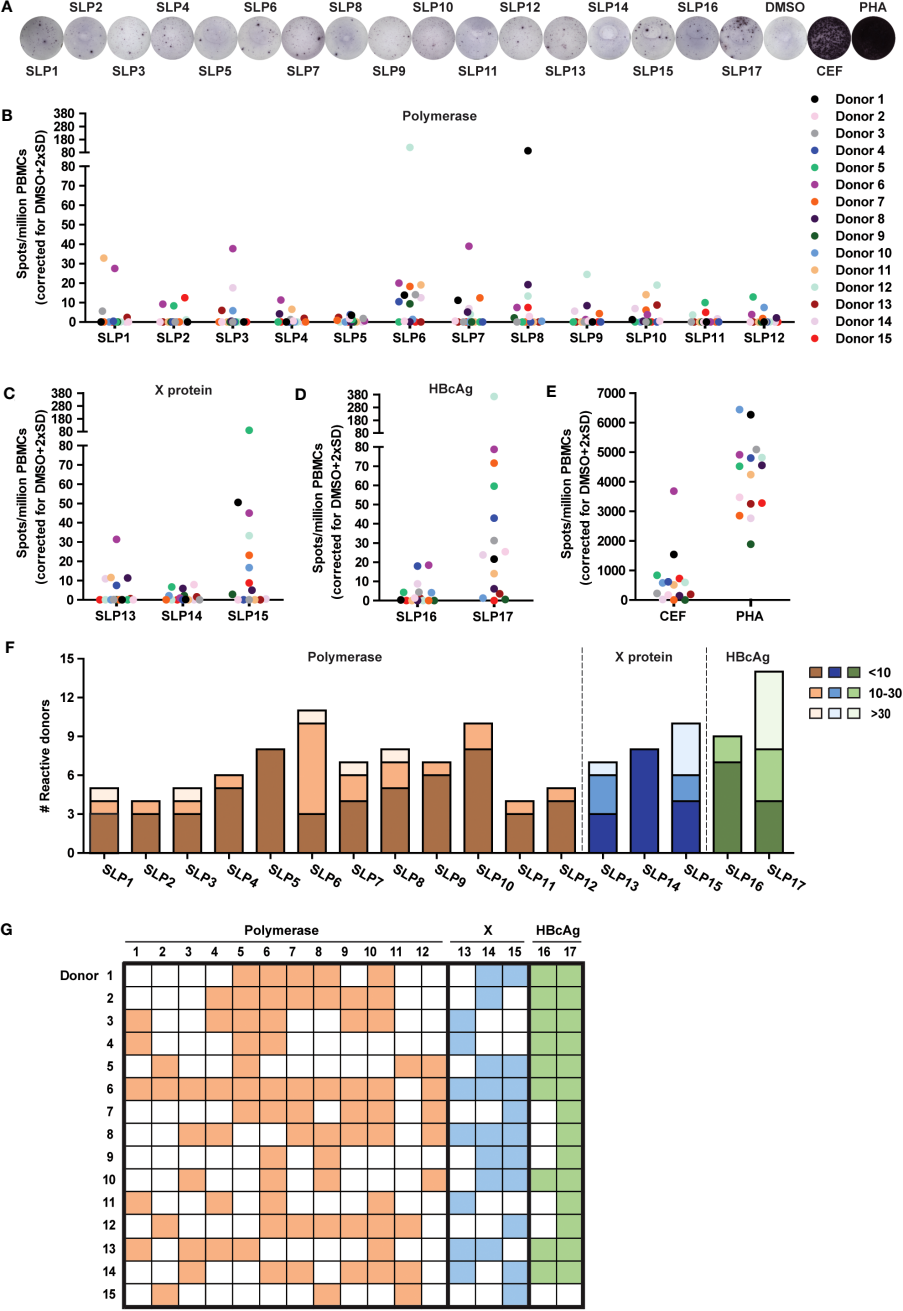
All SLPs elicit an IFNɣ response in PBMCs of several HBV resolvers. The PBMCs of healthy blood donors that resolved an acute HBV infection sometime in their past (rHBV) were stimulated with the 17 SLPs to test their T cell activating capacity using an IFNɣ ELISpot. **(A)** Representative ELISpot images from Donor 6. Summary of ELISpot data for the polymerase **(B)**, X protein **(C)**, HBcAg **(D)**, and SLPs or positive controls **(E)**, being a pool of MHC class I-restricted peptides from human cytomegalovirus, the Epstein–Barr virus, influenza virus (CEF), and phytohaemagglutinin (PHA). Spots are represented as the average number of spots in four wells subtracted by the average number of spots in the DMSO control wells + 2x standard deviation per million PBMCs. Each colored dot represents a different donor (n=15). **(F)** The number of responsive donors per SLP integrated with the strength of the response. Responsive donors with a spot count below 10 are depicted in dark orange (polymerase SLPs), dark blue (X SLPs), or dark green (HBcAg SLPs), spot counts from 10 to 30 are depicted in orange, blue, or green, and spot counts of 30 and above are light orange, light blue, or light green. **(G)** Visual overview of the responses for every rHBV donor to the polymerase (orange), X protein (blue), and HBcAg protein (green) SLPs.

The 17 SLPs were tested for T cell activating potential on PBMCs from 15 healthy blood donors of our local blood bank that had resolved an acute HBV infection in the past (HBV resolver; rHBV). These blood donors all exhibited two or more of the HLA supertypes that the SLP design was based on (**Supplementary Table 2**). T cell activation was assessed by an IFNɣ ELISpot assay on PBMCs. Cryopreserved PBMCs were stimulated with each of the individual SLPs for 22 hours, a length of stimulation favoring the activation of CD8+ T cell responses (34). The average number of observed IFNɣ-producing cells (spots) was corrected for the number of spots observed with the DMSO solvent controls (**Figure 2A**; Methods). Interestingly, all SLPs, regardless of protein origin (**Figures 2B–D**), could elicit an IFNγ response in four or more rHBVs. The magnitude of the response varied for the different SLPs and between individuals and reached up to 365 spots per million PBMCs (SLP17, **Figure 2D**). This variation was also observed in response to the positive controls (**Figure 2E**). The 17 SLPs were also tested on the PBMCs of three healthy controls that had never been infected with hepatitis B. These yielded limited responses to the SLPs (**Supplementary Figure 4A**). A non-relevant SLP, derived from the cancer germline antigen PRAME, yielded responses in 3 out of the 18 healthy donors (15 rHBV, 3 non-HBV exposed; not shown). Based on the average spot counts, an overview of the number of reactive donors including the magnitude of the response was generated (**Figures 2F, G**). Since the frequency of SLP-specific T cells can be expected to be low in the periphery of individuals that cleared an HBV infection, especially if this was long ago, the number of spots can vary between the four wells of a quadruplicate measurement. Using the average spot count could therefore underestimate the response. For this reason, the spot count was also calculated and depicted as cumulative spots in the four wells subtracted by the cumulative spot count in the DMSO control condition (**Supplementary Figures 4B–E**). Taken together, all designed SLPs could induce an IFNɣ response in four or more rHBV donors.

### SLPs can expand PBMCs from both resolvers and chronic HBV patients, but patient PBMCs harbor an altered CD4/CD8 T cell ratio that is maintained by SLP-induced expansion

Based on both the average spot count, the cumulative spot count, number of included epitopes, and covered HLA types, we selected the 10 best performing SLPs for further, more detailed, *ex vivo* testing on PBMCs from both rHBV donors and cHBV patients. This was done because the IFNγ ELISpot does not indicate which cell type within the PBMCs produced the IFNγ; To get more insight on this matter, PBMCs of both rHBV donors (n=6) and cHBV patients (n=5) that were matched based on HLA I type were cultured with two SLP mixes (below) in the presence of IL-2 for 13-14 days in total to induce expansion of the SLP-specific T cells followed by SLP restimulation and flow cytometry staining for T cell identification and activation markers as well as intracellular cytokines. For these experiments, we selected HBeAg negative patients with low serum viral loads (HBV DNA ≤ 1000 IU/mL), serum ALT levels below the upper limit of normal (56 IU/liter), and low to moderate fibrosis (fibroscan F0-F2) (**Table 1**). This patient population in general harbors a population of HBV-specific T cells and, because of their low disease burden, would be candidates to safely receive our SLP vaccine in the future (6).

**Figure 3:**
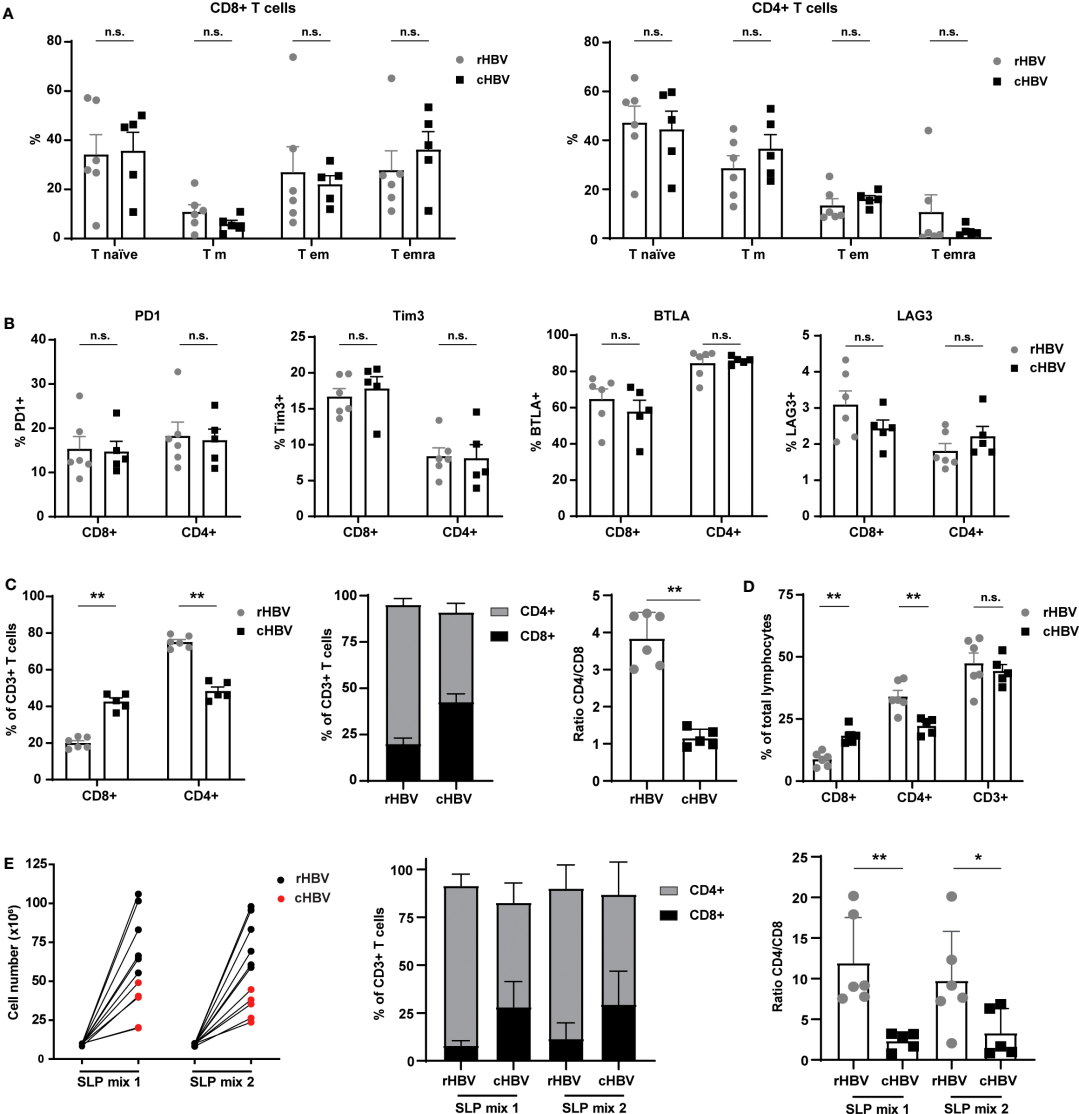
Comparable phenotypes of total T cell population between HBV resolvers and chronic HBV patients, but patients exhibit an altered CD4/CD8 ratio maintained by SLP-induced expansion. PBMCs of HBV resolvers (rHBV, n=6) and chronic HBV patients (cHBV, n=5) were analyzed for the expression of maturation markers and inhibitory molecules by T cells using flowcytometry. **(A)** Based on the expression of CCR7 and CD45RA, four different subsets within the CD4+ and CD8+ T cells were determined; naive T cells (T naive; CCR7+CD45RA+), central memory T cells (T cm; CCR7+CD45RA-), effector memory T cells (T em; CCR7-CD45RA-), and terminally differentiated effector memory T cells (T emra; CCR7-CD45RA+). The subsets are displayed as percentage (%) of the total CD8+ T cell (left) or of the total CD4+ (right) T cell population. **(B)** In a separate staining, the expression of inhibitory markers PD1, Tim3, BTLA, and LAG3 was determined. Expression is depicted as percentage (%) of the total CD8+ T cell (left part of the graphs) or total CD4+ T cell (right part of the graphs) population. **(C)** The percentage (%) of CD8+ and CD4+ cells within the total CD3+ T cell population and their ratio. **(D)** The percentage of CD8+, CD4+, and CD3+ of the total lymphocyte population. **(E)** Live cell numbers by trypan blue (left panel), percentage of CD4+ and CD8+ within the CD3+ T cell population (middle panel), and CD4/CD8 ratio (right panel) in PBMCs expanded for 14 days with two different mixes of SLPs (SLP mix 1 and SLP mix 2) containing 5 SLPs each. N.s., non-significant, *p<0.05, **p<0.01.

T cell maturation/development status and the expression of inhibitory/exhaustion markers between the resolvers and patients could affect responses to the SLPs. For this reason, we first inspected overall T cell phenotypes in the PBMC samples. Based on the expression of CCR7 and CD45RA, no significant differences in frequencies of naive (T naive), effector memory (Tem), central memory (Tcm), and terminally differentiated effector memory (TemRA) subsets were observed between rHBV and cHBV blood donors (**Figure 3A**). However, CD8+ T cells of the TemRA subset appeared more numerous in the PBMCs of many cHBV patients compared with those from rHBV donors. Furthermore, the overall expression of inhibitory markers PD1, Tim3, BTLA, and LAG3 on both CD4+ and CD8+ T cells was not different between the two groups (**Figure 3B**). Interestingly, the percentage of CD8+ and CD4+ cells within the CD3+ T cell population was different between rHBV donors and cHBV patients (**Figure 3C**). PMBCs of rHBV donors contained around 75% CD4+ T cells and 20% CD8+ T cell (out of CD3+ cells), giving a CD4+/CD8+ ratio of nearly 4. PBMCs from cHBV patients, in contrast, displayed almost equal amounts of CD4+ and CD8+ T cells and therefore harbored a much lower CD4/CD8 ratio of nearly 1 (**Figure 3C**). No difference in the percentage of CD3+ T cells of total leukocytes was observed between resolvers and patients, indicating that the lower CD4+/CD8+ ratio was likely due to an actual increase in the numbers of CD8+ T cells at the cost of CD4+ T cells (**Figure 3D**).

Next, we expanded PBMCs from each donor or patient with the best performing SLPs encompassing five polymerase SLPs (SLP5-8 and SLP10), three X protein SLPs (SLP13-15), and two HBcAg SLPs (SLP16 and SLP17). SLPs originating from the same HBV protein were divided over the two SLP pools (SLP Mix 1: SLP5, SLP7, SLP13, SLP15, and SLP17; SLP Mix 2: SLP6, SLP8, SLP10, SLP14, and SLP16). Although to a lower extent compared with rHBV donors, the PBMCs of cHBV patients proliferated in response to both SLP pools (**Figure 3E**). Remarkably, the altered CD4/CD8 ratio was maintained after SLP-induced proliferation. Notably, culture conditions seemed to have favored proliferation of CD4+ T cells within cultures of PBMCS from both cHBV patients and rHBV donors as the CD4/CD8 ratio increased after culture compared to the direct *ex vivo* assay (**Figures 3D, E**). In conclusion, the phenotype of the total T cell population was comparable between cHBV patients and rHBV donors included in our assay apart from a consistently different CD4/CD8 ratio that was maintained after *in vitro* SLP-induced expansion.

### SLPs induce activation of both CD8+ and CD4+ T cells

**Figure 4:**
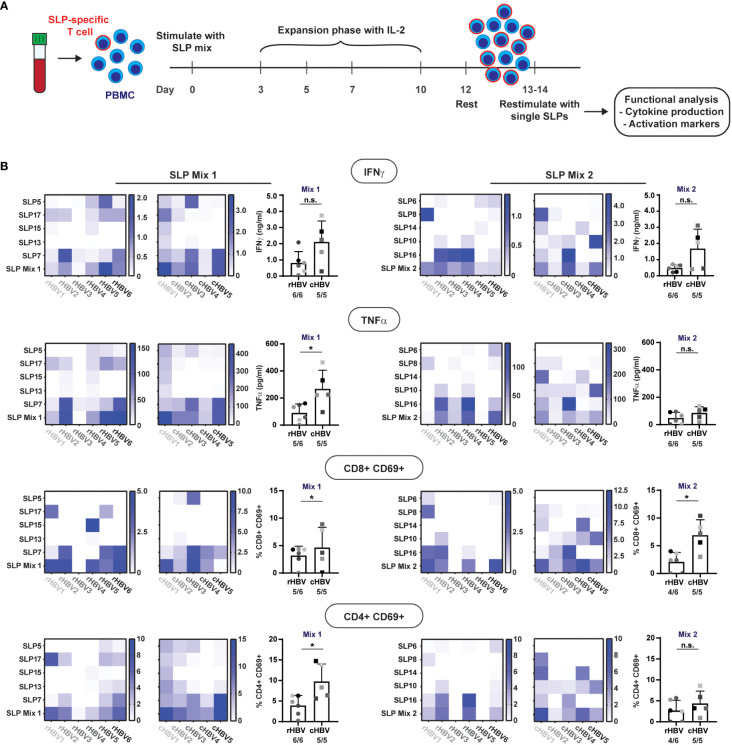
SLPs activate both CD8+ and CD4+ T cells. The 10 best performing SLPs were selected for further analysis and divided over two mixes; SLP Mix 1 and SLP Mix 2.**(A)** Schematic overview of the expansion protocol to expand the SLP-specific T cells (red outline). **(B)** Heat maps were created to visualize the production of IFNɣ and TNFα (upper two rows) in response to restimulation with the different single SLPs and the total SLP mix (left: expanded with SLP Mix 1, right: expanded with SLP Mix 2) for the rHBV (left) and cHBV patients (right). Each tested individual is depicted separately. Values are corrected for background by subtraction of the average value in the negative controls + 2x standard deviation. Next to the heatmaps, the summary of the response to the total SLP mix is depicted including the number of responders out of the total number of tested individuals. An individual was marked responder when the response was positive after background correction. In the lower two rows, the expression of CD69 on either CD8+ T cells or CD4+ T cells after restimulation with the single SLPs or the SLP mix is displayed in the heat maps. Values are corrected for background by subtraction of the DMSO negative control value + 2x the difference between the two DMSO controls. n.s. = non-significant, * p<0.05.

To further discriminate between the activation of CD4+ and CD8+ T cells, we directly (i.e., without cryopreservation) restimulated the SLP-pool expanded PBMCs for 22 hours either with the single pool-contained SLPs or again the total corresponding SLP mix (**Figure 4A**). From this restimulation culture, supernatants were collected for multiplex cytokine analysis while the cells were used for flowcytometric analysis of surface activation markers.

All single SLPs induced a cytokine response indicative of T cell activation (i.e., IFNγ and/or TNFα) by expanded PBMCs from at least one and more often from several donors/patients. Of note, IL-2 was hardly produced and the other cytokines tested were either absent or generally following the pattern of IFNγ and TNFα (not shown). Responses to single SLPs varied per donor/patient, but both pools of five SLPs induced both IFNγ and TNFα in expanded PBMCs from all individuals but one (rHBV3; **Figure 4B** upper two rows, bottom row of heat map; no TNFα response). When directly compared, cytokine responses against SLP pool 1 were higher in cHBV patients than the responses observed in rHBV donors, despite the earlier observed higher proliferation in rHBV donors. The cytokine responses to single SLPs can now be used to derive which SLP(s) was (were) responsible for the observed response to the SLP mix. For example, the IFNɣ response induced by SLP mix 1 in rHBV2 can be explained by responses to both SLP17 and SLP7, while the response in cHBV3 can be explained by responses to SLP5 and SLP7. In rHBV1, the IFNɣ response to SLP mix 2 can solely be ascribed to SLP8, while the observed IFNɣ response in rHBV4 was likely a result of SLP10 and SLP16. The same applies to the observed TNFα response. Interestingly, the pattern of the IFNɣ response is highly similar to the pattern of the TNFα response indicating that both cytokines are often produced together in response to both the SLP mixes or individual SLPs.

To determine whether the observed cytokines in the supernatant were more likely produced by CD8+ or CD4+ T cells, we also inspected activation of these two classes of T cells upon restimulation. Expression of activation marker CD69 was induced on both CD8+ and CD4+ T cells in response to each of the two SLP mixes in the majority of rHBV and cHBV indicating that both CD8+ and CD4+ T cells were activated (**Figure 4B**, lower two rows). Again, and now for both SLP pools, cHBV patient PBMC responses were larger than those observed with cells from rHBV donors. For a few individual SLPs, the predominant activation of either CD4+ T cells (e.g., response to SLP17 in rHBV2) or CD8+ T cells (e.g., response to SLP15 in rHBV4) was observed, but the majority of SLPs induced both CD4+ and CD8+ T cell activation in expanded PBMCs from one or multiple rHBV donors and/or cHBV patients. Similar data were obtained with additional, more cell specific markers of activation: CD137 on CD8+ T cells and CD134 on CD4+ T cells (**Supplementary Figure 5**).

In summary, single SLPs were able to induce activation of CD4+ and/or CD8+ T cells in PBMCs of one or several individuals, while pools of five SLPs could trigger more ubiquitous, multifunctional T cell responses in cells from both resolvers and chronic patients.

### Responses to the same SLP are driven by dissimilar epitopes in different donors

**Figure 5:**
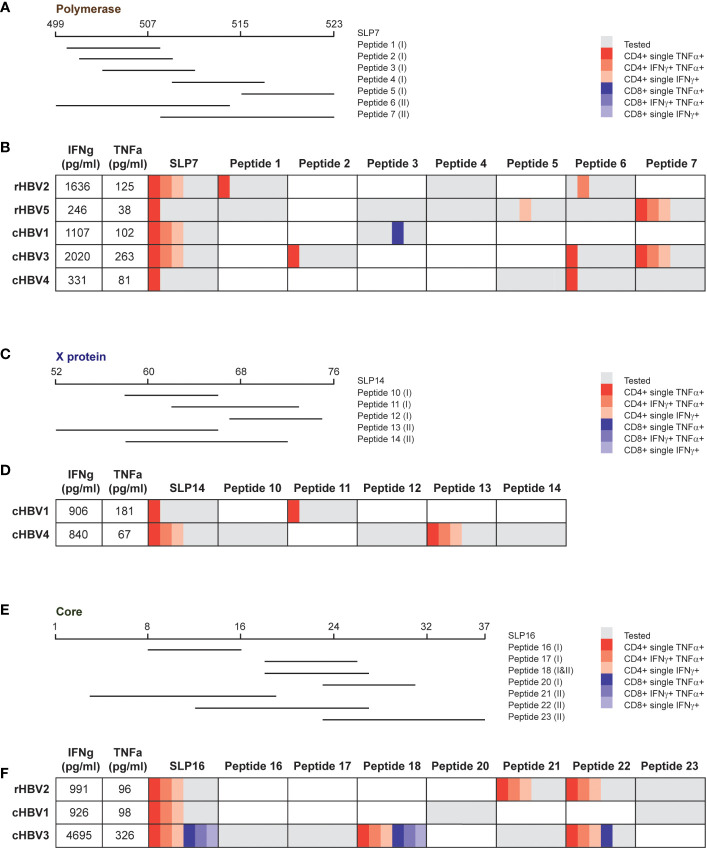
Response to the same SLP can be explained by dissimilar SLP-contained peptides in different donors. For each protein, one SLP was selected to analyze the response in more detail. **(A, C, E)** Overview of the tested peptides for SLP7, SLP14, and SLP16, respectively, for indicated patient and/or resolvers. The HLA class is depicted between brackets for every peptide: (I) HLA I and (II) HLA II. **B, D**, **F**) Overview of the cytokine production by CD4+ and CD8+ T cells after stimulation with the SLP or the peptides for the tested individuals. The first two columns display the production of IFNγ (first column) and TNFα (second column) that was detected via multiplex analysis after restimulation with the respective SLP. The other columns show the results of the intracellular cytokine staining. Gray depicts the peptides that were tested in the respective individuals, which was based on the HLA type of the individual and the (predicted) binding capacity of the peptide. The different red and blue shades indicate single or double cytokine-producing CD4+ and CD8+ T cells, respectively.

For each protein of interest, one SLP was selected for an even more extensive analysis of the observed response to delineate whether the same or different SLP-contained epitopes were driving the response. SLP7 (pol499-523), SLP14 (x52-76), and SLP16 (c1-37) were selected for polymerase, the X protein, and the HBcAg protein, respectively, based on the highest number of contained epitopes and covered HLA types (**Figure 5**). For each SLP, a selection of SLP-contained HLA ligands was synthetized based on previously described and predicted HLA class I and II epitopes and binders (**Figures 5A, C, E**; **Supplementary Table 2**). Cryopreserved PBMCs that had been expanded with the SLP pool containing each of the three SLPs of interest were re-stimulated with either the SLP or the SLP-contained HLA I or II ligands (all separately) for 6 hours followed by an intracellular cytokine staining (ICS) for IFNɣ and TNFα. Each SLP was tested in those individuals, either rHBV or cHBV, who upon the direct re-stimulation after expansion (**Figure 4**) had shown good IFNɣ and TNFα response to the respective SLP by multiplex analysis. Based on the HLA type of the selected individual, the matching set of HLA ligands was tested.

Most of the CD4+ T cell activation observed after full length SLP restimulation of freshly expanded cells (**Figure 4**) was matched by an intracellular CD4+ cytokine response in SLP-restimulated cryopreserved cells (**Figure 5**). In contrast, a CD8+ T cell cytokine response by ICS was only observed for the full length SLP16 in cells from one cHBV patient, indicating that the cryopreservation step or the different restimulation protocol and read-out may have hampered CD8+ T cell activation. Nonetheless, by ICS, the majority of SLP responses could be pinpointed to one or more SLP-contained minimal HLA ligands via the production of IFNɣ, TNFα, or both (**Figures 5B, D, F**). The responsible HLA ligand(s), however, varied between the individuals, reflecting their different HLA types (**Supplementary Table 1**). As an example, the response to SLP7 was likely carried by peptide 1 and 6 in rHBV2, but by peptide 2, 6, and 7 in cHBV3. Further, the response to SLP14 can likely be attributed to peptide 11 in cHBV1, while peptide 13 was the responsible HLA ligand in cHBV4. Like the full length SLPs, the minimal HLA ligands also mainly induced CD4+ T cell responses apart from one peptide in SLP7 and two peptides in SLP16. The latter two were minimal HLA I and HLA II ligands that both contained the well-established HBcAg_18-27_ CD8+ and CD4+ T cell epitope (**Figure 5F**). These results indicate that the response to single HBV-based SLPs is driven by distinct HLA ligands in different individuals illustrating that not only the combination of multiple SLPs, but also single SLPs contribute to achieving broad population coverage.

## Discussion

In this study, we have taken the next step towards a globally applicable therapeutic vaccine for chronic HBV to be used in a multi-therapeutic treatment regimen to cure HBV. By focusing on regions within the pol, HBcAg, and X proteins that were conserved across HBV genotypes and rich in epitopes for globally prevalent HLA types, we generated 17 SLPs that are now strong potential candidates for inclusion in an SLP-based vaccine for clinical evaluation.

All 17 SLPs were able to trigger and/or revive T cell responses in multiple healthy individuals that express a heterogenous set of HLA types and that had resolved HBV at an unknown moment in their past. Healthy donors not previously exposed to HBV showed limited responses but were not fully unresponsive. More individuals need to be tested to draw any firm conclusions on the responsiveness of unexposed individuals to the 17 SLPs. However, these unexpected responses in HBV-naive individuals may be explained by high precursor frequencies of HBV-reactive naive T cells as was observed for several HBV epitopes by Cheng and co-workers (20), and/or may represent heterologous T cells recognizing epitopes from other pathogens/protein sources similar in peptide sequence or 3D peptide-HLA structure (35).

Although each of the 17 SLPs could still be considered a candidate for therapeutic vaccine inclusion, we focused in more detail on the 10 best performing SLPs by ELISpot in the 15 rHBV donors. We found that these 10 SLPs activated both CD4+ and CD8+ T cells in blood leukocytes extracted from both resolvers and chronically infected individuals. Each SLP triggered responses in a different set of individuals and importantly pooling of five SLPs in two separate mixes yielded responses in nearly all resolvers and patients. Together these two pools of 10 SLPs even fully covered all individuals. Typically, SLP-based vaccines harbor 10-20 SLPs, indicating that achieving significant population coverage is within reach (14, 36, 37). To place this high *in vitro* success rate further into perspective, the ethnicities, viral genotypes, and HLA types within our cohort are important to consider. Unfortunately, the ethnicities of our resolver cohort were not available to us. Based on geographic location, it is likely that this cohort was dominated by Caucasian participants and that this cohort had been infected with viral genotypes A and D that are most prevalent in the Netherlands (38). HLA types, however, were heterogenous in the resolver cohort and covered each of the six aforementioned prevalent HLA supertypes (A1, A2, A3, A24, B7, and B8) including also some of the HLA subtypes most prevalent in Asia (i.e., A11 and A24), where cHBV prevalence is high (39). The cHBV patient cohort was heterogeneous and included five out of six prevalent HLA types, four viral genotypes, and both Caucasian as well as Asian participants. Further clinical development and immunomonitoring of SLPs will reveal the true ability of each SLP and of a therapeutic vaccine comprised of multiple HBV SLPs to induce T cell responses across ethnicities, genotypes, and HLA types.

For a selection of three SLPs from each viral protein, we zoomed in on the specific minimal HLA ligands driving the SLP-induced T cell activation. This information is useful also to derive the epitope-specific T cell responses of interest to follow up on in clinical studies testing the *in vivo* immunogenicity of the SLPs. Experiments demonstrated that SLP-induced T cell responses in different individuals were driven by distinct HLA epitopes, underscoring the ability of SLPs to act across HLA boundaries. Some SLP-driven T cell responses could not be explained by any of the minimal HLA ligands included in our re-stimulation assay. This may have been a result of the stronger CD4-bias in restimulation experiments after cryopreservation of SLP-expanded PBMCs and/or caused by incomplete coverage of HLA ligands due to imperfect *in silico* HLA binding prediction. It is noteworthy that, despite the observed CD4 bias, the immunodominant HBcAg_18-27-_ epitope induced a potent CD4+ and CD8+ response in one cHBV patient and was even cross-presented to CD8+ T cells from longer peptides (i.e., the full length SLP and the longer HLA II ligand) by the cryopreserved expanded PBMCs.

Our experiments indicate that designed SLPs are able to induce broad immune responses across the population. Because HLA I ligands are cross-presented more efficiently from SLPs than from whole protein, SLP-based vaccines may broaden T cell responses by enhancing T cells that recognize subdominant epitopes (11, 12). Such subdominant epitopes may not be cross presented well naturally *in vivo* from whole viral proteins during ongoing viral infection. Specific stimulation of subdominant conserved epitopes in mice and chimpanzees has been demonstrated to broaden T cell responses and to induce T cells that hold potential to contain viral infections (40–42).

Counterintuitively, SLPs induced T cell activation and cytokine production when using cells from cHBV patients rather than from resolvers. This could be partially explained by the fact that we used cells from individuals with low viral burden and/or on antiviral treatment. This patient population has been associated with the largest numbers and least dysfunctional T cells for the antigens targeted by our SLPs (20, 22, 43, 44). Furthermore, antigen exposure in chronic patients was likely more recent and, importantly, patients were younger than resolvers [cHBV: 42.6 (+/- 13.6), rHBV: 60.2 (+/- 7.3) (**Table 1**)]. These factors may together have rendered HBV-specific T cells more numerous in the peripheral blood and/or may have enhanced responses.

Despite the high responses of patient blood cells to the SLPs, we did observe some signs of immune dysfunction in our cohort of cHBV patients. Most apparent was the lower expansion of total leukocytes in the presence of SLP pools and a much lower CD4+/CD8+ T cell ratio in cHBV patients compared with rHBV donors. A low blood CD4+/CD8+ T cell ratio has been reported before for HBV, but observations were not always consistent or accompanied by matched healthy controls (45–47). The small number of patients included in our study was too small and heterogeneous to shed any light on this matter and, importantly, also in our study, the healthy HBV resolvers were not matched to patients in terms of age and gender. Older females, who mostly comprised our resolver cohort, are especially known to have relatively more peripheral CD4+ T cells and less CD8+ T cells (48). Thus, both a lower CD4+/CD8+ ratio in the studied cHBV patients and a higher ratio in studied rHBV donors may have contributed to the observed ratio difference. For this reason, it might be of interest to monitor the CD4+/CD8+ ratio in future clinical studies on therapeutic vaccines in HBV. Nevertheless, the *ex vivo* T cell expansion data show that HBV-specific T cells can be expanded by SLP stimulation even in cHBV individuals. In addition, SLP vaccination cannot only be considered to restore established but exhausted/weak HBV-specific T cell responses, but also to elicit *de novo* T cell responses to these and not yet utilized HBV T cell epitopes.

Taken together, we have designed a set of novel SLPs capable of inducing CD4+ helper and CD8+ cytotoxic T cell responses in both HBV resolvers and chronic HBV patients targeting conserved viral epitopes not easily evaded by viral mutation. These abilities endow these new SLPs with high potential to induce effective long-lasting antiviral T cell responses and render them candidates to be included in a globally applicable therapeutic HBV vaccine. By current consensus, a stand-alone therapy of any HBV-directed therapeutic vaccine will likely not suffice (5, 6). A heterologous prime-boost with a vaccine also inducing HBV-directed antibody responses may be required (reviewed in (6)). Furthermore, to combat the immune exhaustion and suppression that results from long-term chronic viral infection and/or directly from the virus itself, combination with other forms of therapy is likely needed. Examples include NA therapy and siRNA prior to vaccine administration to reduce viral load and to diminish viral protein expression, respectively, or T cell-enhancing drugs (e.g., checkpoint inhibitors and metabolism modifiers) to combat T cell dysfunction (49–53). Such combinations have already proven their value alongside SLPs or other forms of therapeutic vaccines in cancer immunotherapies (13, 14, 54). Design of the ultimate combination regime capable of establishing a functional cure for cHBV starts with the creation of highly immunogenic vaccines and by obtaining detailed insight into the strength, quality, and dynamics of vaccine responsive T cells *in vivo*. The outcome of monotherapy vaccination in cHBV individuals will dictate the design of subsequent clinical combinations with an anti-viral drug likely needed to effectively clear the virus.

## Data availability statement

The original contributions presented in the study are included in the article/**supplementary files**, further inquiries can be directed to the corresponding author/s.

## Ethics statement

The study involved humans and was approved by Medical Ethics Committee of the Erasmus MC University Medical Center Rotterdam. The study was conducted in accordance with the local legislation and institutional requirements. The participants provided their written informed consent to participate in this study.

## Author contributions

Study concept and design: SB, DJ, MB, CM, WK, RB, and RM; acquisition of data; DJ, RL, KK, AW, AK, and RP; analysis and interpretation of data; DJ, SB, KK, AW, WK, and CM; drafting of the manuscript: DJ and SB; critical revision of the manuscript for important intellectual content: AW, CM, AK, and RP; statistical analysis; DJ, KK, and AW; obtained funding; SB, WK, RM, and CM; study supervision: SB. All authors contributed to the article and approved the submitted version.

## References

[1] Schweitzer A Horn J Mikolajczyk RT Krause G Ott JJ. Estimations of worldwide prevalence of chronic hepatitis B virus infection: a systematic review of data published between 1965 and 2013. Lancet (2015) 386:1546–55. doi: 10.1016/S0140-6736(15)61412-X 26231459

[2] Lampertico P Agarwal K Berg T Buti M Janssen HLA Papatheodoridis G. EASL 2017 Clinical Practice Guidelines on the management of hepatitis B virus infection. J Hepatol (2017) 67:370–98. doi: 10.1016/j.jhep.2017.03.021 28427875

[3] Cornberg M Lok AS-F Terrault NA Zoulim F Berg T Brunetto MR. Guidance for design and endpoints of clinical trials in chronic hepatitis B - Report from the 2019 EASL-AASLD HBV Treatment Endpoints Conference‡. J Hepatol (2020) 72:539–57. doi: 10.1016/j.jhep.2019.11.003 31730789

[4] Fanning GC Zoulim F Hou J Bertoletti A. Therapeutic strategies for hepatitis B virus infection: towards a cure. Nat Rev Drug Discovery (2019) 18:827–44. doi: 10.1038/s41573-019-0037-0 31455905

[5] Maini MK Pallett LJ. Defective T-cell immunity in hepatitis B virus infection: why therapeutic vaccination needs a helping hand. Lancet Gastroenterol Hepatol (2018) 3:192–202. doi: 10.1016/S2468-1253(18)30007-4 29870733

[6] Jansen DTSL Dou Y de Wilde JW Woltman AM Buschow SI. Designing the next-generation therapeutic vaccines to cure chronic hepatitis B: focus on antigen presentation, vaccine properties and effect measures. Clin Transl Immunol (2021) 10. doi: 10.1002/cti2.1232 PMC7809700 33489122

[7] Ahrends T Spanjaard A Pilzecker B Bąbała N Bovens A Xiao Y. CD4+ T cell help confers a cytotoxic T cell effector program including coinhibitory receptor downregulation and increased tissue invasiveness. Immunity (2017) 47:848–861.e5. doi: 10.1016/j.immuni.2017.10.009 29126798

[8] Asabe S Wieland SF Chattopadhyay PK Roederer M Engle RE Purcell RH. The size of the viral inoculum contributes to the outcome of hepatitis B virus infection † Downloaded from. J Virol (2009) 83:9652–62. doi: 10.1128/JVI.00867-09 PMC2748002 19625407

[9] Thimme R Wieland S Steiger C Ghrayeb J Reimann KA Purcell RH. CD8+ T cells mediate viral clearance and disease pathogenesis during acute hepatitis B virus infection. J Virol (2003) 77:68–76. doi: 10.1128/jvi.77.1.68-76.2003 12477811 PMC140637

[10] Melief CJM Van Hall T Arens R Ossendorp F van der Burg SH. Therapeutic cancer vaccines. J Clin Invest (2015) 125:3401–12. doi: 10.1172/JCI80009 PMC4588240 26214521

[11] Rosalia RA Quakkelaar ED Redeker A Khan S Camps M Drijfhout JW. Dendritic cells process synthetic long peptides better than whole protein, improving antigen presentation and T-cell activation. Eur J Immunol (2013) 43:2554–65. doi: 10.1002/eji.201343324 23836147

[12] Zhang H Hong H Li D Ma S Di Y Stoten A. Comparing pooled peptides with intact protein for accessing cross-presentation pathways for protective CD8+ and CD4+ T cells. J Biol Chem (2009) 284:9184–91. doi: 10.1074/jbc.M809456200 PMC2666570 19193636

[13] Massarelli E William W Johnson F Kies M Ferrarotto R Guo M. Combining immune checkpoint blockade and tumor-specific vaccine for patients with incurable human papillomavirus 16–related cancer. JAMA Oncol (2019) 5:67. doi: 10.1001/jamaoncol.2018.4051 30267032 PMC6439768

[14] Ott PA Hu Z Keskin DB Shukla SA Sun J Bozym DJ. An immunogenic personal neoantigen vaccine for patients with melanoma. Nature (2017) 547:217–21. doi: 10.1038/nature22991 PMC5577644 28678778

[15] Zom GGP Khan S Filippov DV Ossendorp F. TLR ligand-peptide conjugate vaccines. Toward clinical application. Adv Immunol (2012) 114:177–201. doi: 10.1016/B978-0-12-396548-6.00007-X 22449782

[16] Dou Y Van Montfoort N Van Den Bosch A De Man RA Zom GG Krebber WJ. HBV-derived synthetic long peptide can boost CD4 + and CD8 + T-cell responses in chronic HBV patients ex vivo. J Infect Dis (2018) 217:827–39. doi: 10.1093/infdis/jix614 PMC5853453 29220492

[17] Schuch A Salimi Alizei E Heim K Wieland D Kiraithe MM Kemming J. Phenotypic and functional differences of HBV core-specific versus HBV polymerase-specific CD8+ T cells in chronically HBV-infected patients with low viral load. Gut (2019) 68:905–15. doi: 10.1136/gutjnl-2018-316641 30622109

[18] Hoogeveen RC Robidoux MP Schwarz T Heydmann L Cheney JA Kvistad D. Phenotype and function of HBV-specific T cells is determined by the targeted epitope in addition to the stage of infection. Gut (2019) 68:893–904. doi: 10.1136/gutjnl-2018-316644 30580250

[19] Boni C Fisicaro P Valdatta C Amadei B Di Vincenzo P Giuberti T. Characterization of hepatitis B virus (HBV)-specific T-cell dysfunction in chronic HBV infection. J Virol (2007) 81:4215–25. doi: 10.1128/JVI.02844-06 PMC1866111 17287266

[20] Cheng Y Zhu YO Becht E Aw P Chen J Poidinger M. Multifactorial heterogeneity of virus-specific T cells and association with the progression of human chronic hepatitis B infection. Sci Immunol (2019) 4:eaau6905. doi: 10.1126/sciimmunol.aau6905 30737354

[21] Rivino L Le Bert N Gill US Kunasegaran K Cheng Y Tan DZM. Hepatitis B virus–specific T cells associate with viral control upon nucleos(t)ide-analogue therapy discontinuation. J Clin Invest (2018) 128:668–81. doi: 10.1172/JCI92812 PMC5785266 29309050

[22] Le Bert N Gill US Hong M Kunasegaran K Tan DZM Ahmad R. Effects of hepatitis B surface antigen on virus-specific and global T cells in patients with chronic hepatitis B virus infection. Gastroenterology (2020) 159:652–64. doi: 10.1053/j.gastro.2020.04.019 32302614

[23] Boni C Laccabue D Lampertico P Giuberti T Viganò M Schivazappa S. Restored function of HBV-specific T cells after long-term effective therapy with nucleos(t)ide analogues. Gastroenterology (2012) 143:963–973.e9. doi: 10.1053/j.gastro.2012.07.014 22796241

[24] Dunn C Peppa D Khanna P Nebbia G Jones M Brendish N. Temporal analysis of early immune responses in patients with acute hepatitis B virus infection. Gastroenterology (2009) 137:1289–300. doi: 10.1053/j.gastro.2009.06.054 19591831

[25] Lucifora J Arzberger S Durantel D Belloni L Strubin M Levrero M. Hepatitis B virus X protein is essential to initiate and maintain virus replication after infection. J Hepatol (2011) 55:996–1003. doi: 10.1016/J.JHEP.2011.02.015 21376091

[26] de Beijer MTA Jansen DTSL Dou Y van Esch WJE Mok JY Maas MJP. Discovery and selection of hepatitis B virus-derived T cell epitopes for global immunotherapy based on viral indispensability, conservation, and HLA-binding strength. J Virol (2020) 94. doi: 10.1128/jvi.01663-19 PMC7081907 31852786

[27] Kunert A Basak EA Hurkmans DP Balcioglu HE Klaver Y van Brakel M. CD45RA+CCR7– CD8 T cells lacking co-stimulatory receptors demonstrate enhanced frequency in peripheral blood of NSCLC patients responding to nivolumab. J Immunother Cancer (2019) 7:149. doi: 10.1186/s40425-019-0608-y 31176366 PMC6555948

[28] Sidney J Peters B Frahm N Brander C Sette A. HLA class I supertypes: A revised and updated classification. BMC Immunol (2008) 9:1. doi: 10.1186/1471-2172-9-1 18211710 PMC2245908

[29] Hayer J Jadeau F Deléage G Kay A Zoulim F Combet C. HBVdb: a knowledge database for Hepatitis B Virus. Nucleic Acids Res (2013) 41:566–70. doi: 10.1093/NAR/GKS1022 PMC3531116 23125365

[30] Lumley S Noble H Hadley MJ Callow L Malik A Chua YY. Hepitopes: A live interactive database of HLA class I epitopes in hepatitis B virus. Wellcome Open Res (2016) 1:9. doi: 10.12688/wellcomeopenres.9952.1 27976751 PMC5142601

[31] Chen B Khodadoust MS Olsson N Wagar LE Fast E Liu CL. Predicting HLA class II antigen presentation through integrated deep learning. Nat Biotechnol (2019) 37:1332–43. doi: 10.1038/s41587-019-0280-2 PMC7075463 31611695

[32] Sturniolo T Bono E Ding J Raddrizzani L Tuereci O Sahin U. Generation of tissue-specific and promiscuous HLA ligand databases using DNA microarrays and virtual HLA class II matrices. Nat Biotechnol (1999) 17(6):555–61. doi: 10.1038/9858 10385319

[33] Müller M Gfeller D Coukos G Bassani-Sternberg M. “Hotspots” of antigen presentation revealed by human leukocyte antigen ligandomics for neoantigen prioritization. Front Immunol (2017) 8:1367/BIBTEX. doi: 10.3389/FIMMU.2017.01367/BIBTEX 29104575 PMC5654951

[34] Singh SK Meyering M Ramwadhdoebe TH Stynenbosch LFM Redeker A Kuppen PJK. The simultaneous ex vivo detection of low-frequency antigen-speciWc CD4+ and CD8+ T-cell responses using overlapping peptide pools. Cancer Immunol Immunother (2012) 61:1953–63. doi: 10.1007/S00262-012-1251-3/FIGURES/6 PMC3493661 22491788

[35] Sewell AK. Why must T cells be cross-reactive? Nat Rev Immunol (2012) 12:669. doi: 10.1038/NRI3279 22918468 PMC7097784

[36] van Poelgeest MI Welters MJ Vermeij R Stynenbosch LF Loof NM Berends-van der Meer DM. Vaccination against oncoproteins of HPV16 for noninvasive vulvar/vaginal lesions: lesion clearance is related to the strength of the T-cell response. Clin Cancer Res (2016) 22:2342–50. doi: 10.1158/1078-0432.CCR-15-2594 26813357

[37] Leffers N Lambeck AJ Gooden MJ Hoogeboom BN Wolf R Hamming IE. Immunization with a P53 synthetic long peptide vaccine induces P53-specific immune responses in ovarian cancer patients, a phase II trial. Int J Cancer (2009) 125:2104–13. doi: 10.1002/ijc.24597 19621448

[38] Velkov S Ott JJ Protzer U Michler T. The global hepatitis B virus genotype distribution approximated from available genotyping data. Genes (Basel) (2018) 9. doi: 10.3390/GENES9100495 PMC6210291 30326600

[39] Sette A Sidney J. Nine major HLA class I supertypes account for the vast preponderance of HLA-A and -B polymorphism. Immunogenetics (1999) 50:201–12. doi: 10.1007/s002510050594 10602880

[40] Im EJ Hong JP Roshorm Y Bridgeman A Létourneau S Liljeström P. Protective efficacy of serially up-ranked subdominant CD8+ T cell epitopes against virus challenges. PloS Pathog (2011) 7:e1002041. doi: 10.1371/JOURNAL.PPAT.1002041 21625575 PMC3098219

[41] Donnison T von Delft A Brown A Swadling L Hutchings C Hanke T. Viral vectored hepatitis C virus vaccines generate pan-genotypic T cell responses to conserved subdominant epitopes. Vaccine (2020) 38:5036–48. doi: 10.1016/j.vaccine.2020.05.042 32532545

[42] Steffensen MA Pedersen LH Jahn ML Nielsen KN Christensen JP Thomsen AR. Vaccine Targeting of Subdominant CD8 + T Cell Epitopes Increases the Breadth of the T Cell Response upon Viral Challenge, but May Impair Immediate Virus Control. J Immunol (2016) 196:2666–76. doi: 10.4049/JIMMUNOL.1502018/-/DCSUPPLEMENTAL 26873995

[43] Boni C Penna A Bertoletti A Lamonaca V Rapti I Missale G. Transient restoration of anti-viral T cell responses induced by lamivudine therapy in chronic hepatitis B. J Hepatol (2003) 39:595–605. doi: 10.1016/S0168-8278(03)00292-7 12971971

[44] Park JJ Wong DK Wahed AS Lee WM Feld JJ Terrault N. Hepatitis B virus-specific and global T-cell dysfunction in chronic hepatitis B. Gastroenterology (2016) 150:684–695e5. doi: 10.1053/j.gastro.2015.11.050 26684441 PMC4766024

[45] You J Zhuang L Zhang YF Chen HY Sriplung H Geater A. Peripheral T-lymphocyte subpopulations in different clinical stages of chronic HBV infection correlate with HBV load. World J Gastroenterol (2009) 15:3382. doi: 10.3748/WJG.15.3382 19610139 PMC2712899

[46] Pham BN Mosnier JF Walker F Njapoum C Bougy F Degott C. Flow cytometry CD4+/CD8+ ratio of liver-derived lymphocytes correlates with viral replication in chronic hepatitis B. Clin Exp Immunol (1994) 97:403. doi: 10.1111/J.1365-2249.1994.TB06102.X 7915977 PMC1534853

[47] Sprengers D van der Molen RG Kusters JG Hansen B Niesters HGM Schalm SW. Different composition of intrahepatic lymphocytes in the immune-tolerance and immune-clearance phase of chronic hepatitis B. J Med Virol (2006) 78:561–8. doi: 10.1002/jmv.20576 16555293

[48] Klein SL Flanagan KL. Sex differences in immune responses. Nat Rev Immunol (2016) 16:626–38. doi: 10.1038/nri.2016.90 27546235

[49] Boni C Janssen HLA Rossi M Yoon SK Vecchi A Barili V. Combined GS-4774 and tenofovir therapy can improve HBV-specific T-cell responses in patients with chronic hepatitis. Gastroenterology (2019) 157:227–241.e7. doi: 10.1053/j.gastro.2019.03.044 30930022

[50] Michler T Kosinska AD Festag J Bunse T Su J Ringelhan M. Knockdown of virus antigen expression increases therapeutic vaccine efficacy in high-titer hepatitis B virus carrier mice. Gastroenterology (2020) 158:1762–1775.e9. doi: 10.1053/j.gastro.2020.01.032 32001321

[51] Dembek C Protzer U Roggendorf M. Overcoming immune tolerance in chronic hepatitis B by therapeutic vaccination. Curr Opin Virol (2018) 30:58–67. doi: 10.1016/J.COVIRO.2018.04.003 29751272

[52] Wooddell CI Yuen M-F Chan HL-Y Gish RG Locarnini SA Chavez D. RNAi-based treatment of chronically infected patients and chimpanzees reveals that integrated hepatitis B virus DNA is a source of HBsAg. Sci Transl Med (2017) 9. doi: 10.1126/scitranslmed.aan0241 PMC5830187 28954926

[53] Fisicaro P Barili V Montanini B Acerbi G Ferracin M Guerrieri F. Targeting mitochondrial dysfunction can restore antiviral activity of exhausted HBV-specific CD8 T cells in chronic hepatitis B. Nat Med (2017) 23:327–36. doi: 10.1038/nm.4275 28165481

[54] Sahin U Derhovanessian E Miller M Kloke B-P Simon P Löwer M. Personalized RNA mutanome vaccines mobilize poly-specific therapeutic immunity against cancer. Nature (2017) 547:222–6. doi: 10.1038/nature23003 28678784

