# Supplementary material for: Induction of broad multifunctional CD8+ and CD4+ T cells by hepatitis B virus antigen-based synthetic long peptides ex vivo
Source: Front Immunol. 2023 Sep 13;14:1163118. doi: 10.3389/fimmu.2023.1163118 (PMC10534072; doi:10.3389/fimmu.2023.1163118)
Supplement: Supplementary file 1 [file DataSheet_1.pdf]

SUPPLEMENTARY FIGURES AND TABLE

Induction of broad multifunctional CD8+ and CD4+ T cells by hepatitis B virus antigen-based synthetic long peptides ex vivo

Diahann T.S.L. Jansen[1,#], Monique T.A. de Beijer[1,$], Robbie J. Luijten[1], Kitty Kwappenberg[2], Anna-Sophia Wiekmeijer[2], Amy L. Kessler[1], Roel F.A. Pieterman[1], Rachid Bouzid[1], Willem-Jan Krebber[2], Robert A. de Man[1], Cornelis J.M. Melief[2] and Sonja I. Buschow[1]

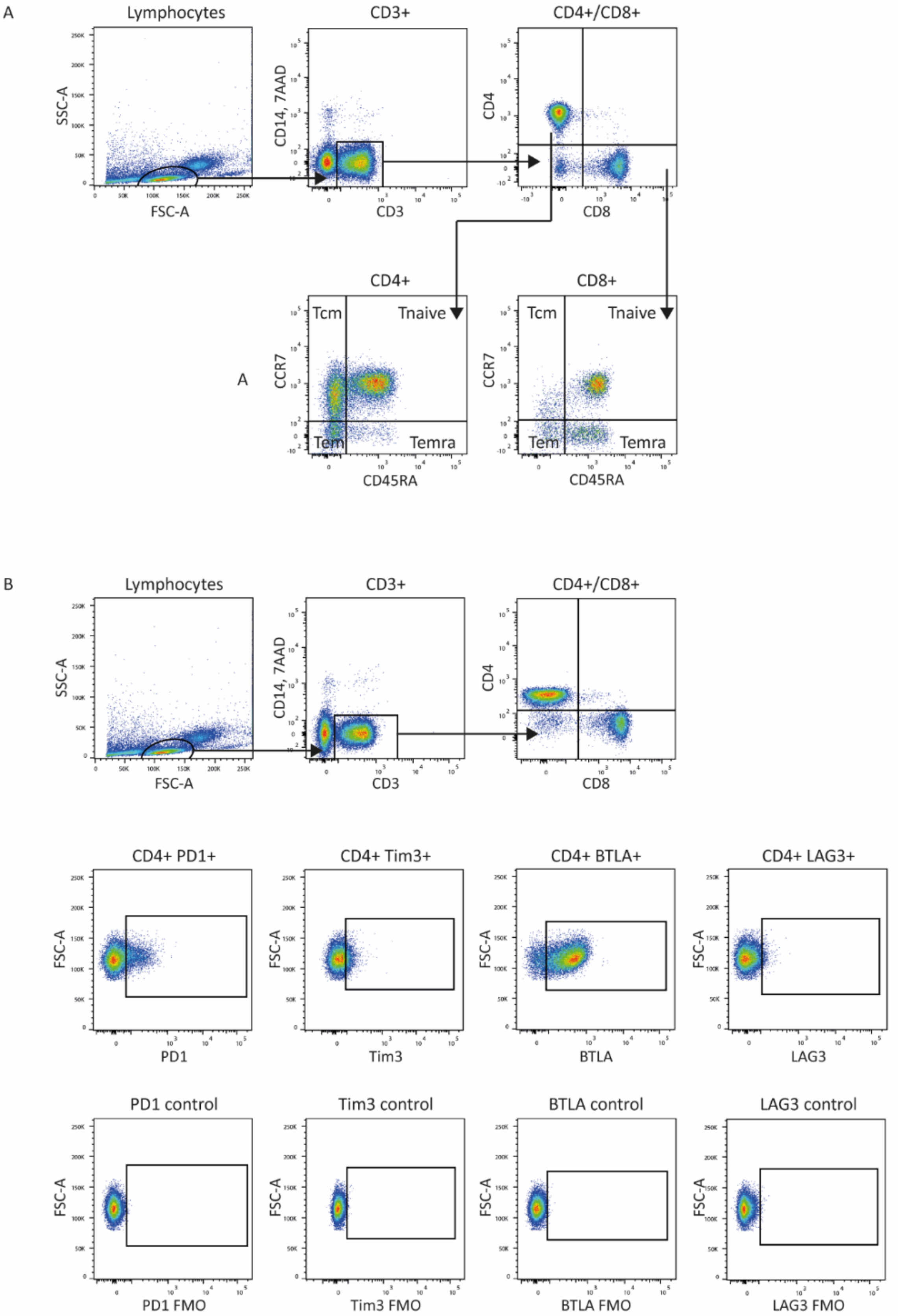
A
Lymphocytes
SSC-A
FSC-A
CD3+
CD14, 7AAD
CD3
CD4+/CD8+
CD4
CD8
CD4+
Tcm
Tnaive
Tem
Temra
CCR7
CD45RA
CD8+
Tcm
Tnaive
Tem
Temra
CCR7
CD45RA
A
B
Lymphocytes
SSC-A
FSC-A
CD3+
CD14, 7AAD
CD3
CD4+/CD8+
CD4
CD8
CD4+ PD1+
FSC-A
PD1
CD4+ Tim3+
FSC-A
Tim3
CD4+ BTLA+
FSC-A
BTLA
CD4+ LAG3+
FSC-A
LAG3
PD1 control
FSC-A
PD1 FMO
Tim3 control
FSC-A
Tim3 FMO
BTLA control
FSC-A
BTLA FMO
LAG3 control
FSC-A
LAG3 FMO

Supplementary figure 1. Gating strategy maturation and inhibitory markers on total T cell population

The expression of maturation and inhibitory markers by the total T cell population of cHBV and rHBV was determined by flowcytometry. Depicted is the gating strategy of a representative donor. A) For the T cell maturation subsets, first, the total lymphocyte population was gated followed by the CD3+ T cell population that was alive and non-monocyte (CD14-7AAD-CD3+). From the CD3+ T cell population CD4+ and CD8+ T cells were gated and subsequently the different maturation subsets were gated for both CD4+ and CD8+; Tem (CCR7-CD45RA-), Tcm (CCR7+CD45RA-), Tmaive (CCR7+CD45RA+) and Temra (CCR7-CD45RA+). B) For the inhibitory markers CD4+ and CD8+ T cells were gated as in A and subsequently the inhibitory markers PD1 (CD279), Tim3 (CD336), BTLA (CD272) and LAG3 (CD223) were plotted against the forward scatter to determine positivity using fluorescence minus one (FMO) controls (bottom row). As example the gating strategy for the CD4+ T cells only is depicted.

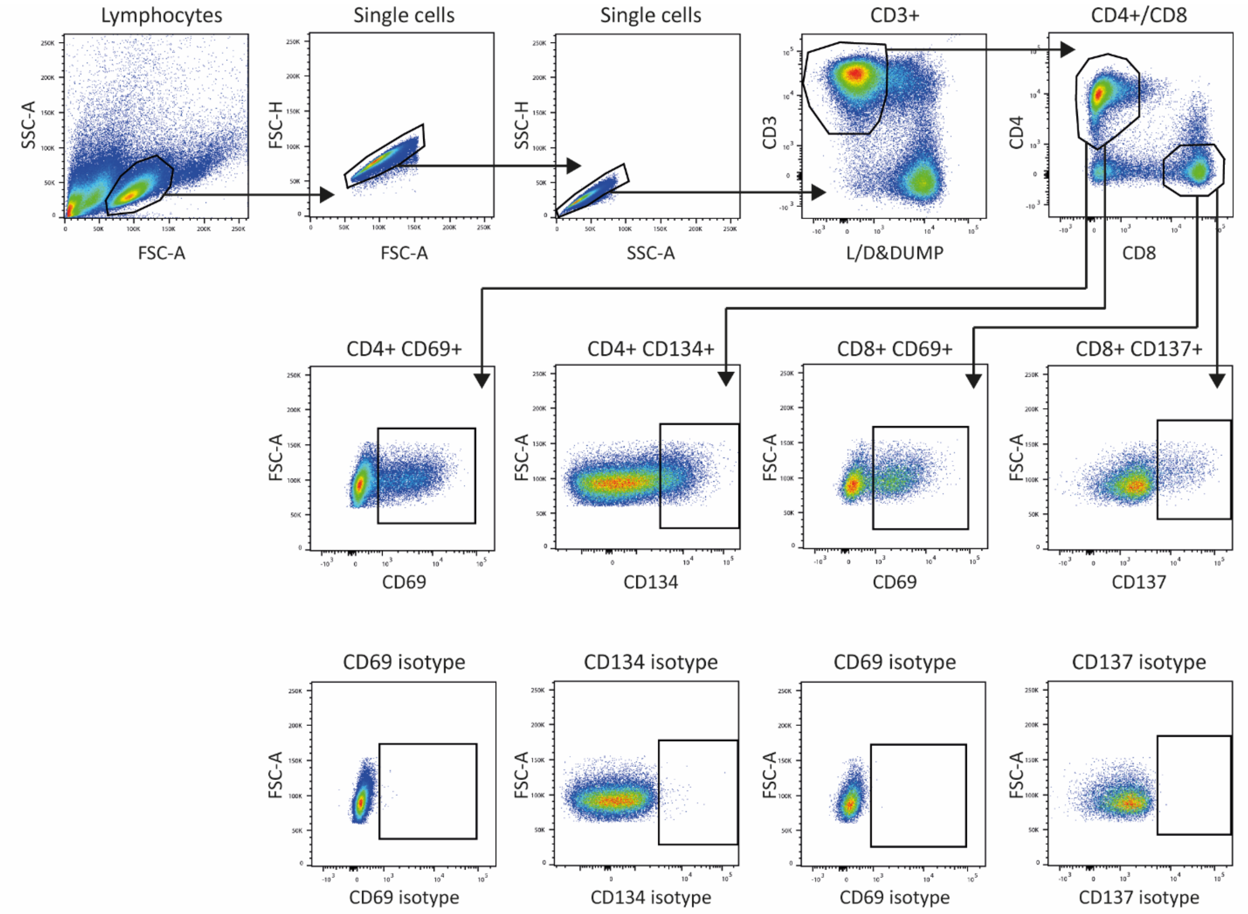

Supplementary figure 2. Gating strategy activation markers after SLP-induced expansion

After expansion with SLP pools and restimulation with either the corresponding SLP pool, the single SLPs or DMSO only, the cells were stained for the following activation markers; CD69, CD134, and CD137. First, the total lymphocyte population was gated followed by selection of single cells using both the forward and side scatter. The CD3+ T cells were identified by gating on the CD3+ LIVE/DEAD-Dump- population, where the LIVE/DEAD and Dump channel markers CD14, CD19 and CD56 were combined in the same channel. Subsequently, CD4+ and CD8+ T cells were gated following identification of CD4+ CD69+, CD4+ CD134+, CD8+ CD69+ and CD8+ CD137+ T cells using isotype control staining (bottom row).

Values in the heatmap in Figure 4 are the percentages after SLP stimulation corrected for the percentages in the DMSO control samples + 2x standard deviation.

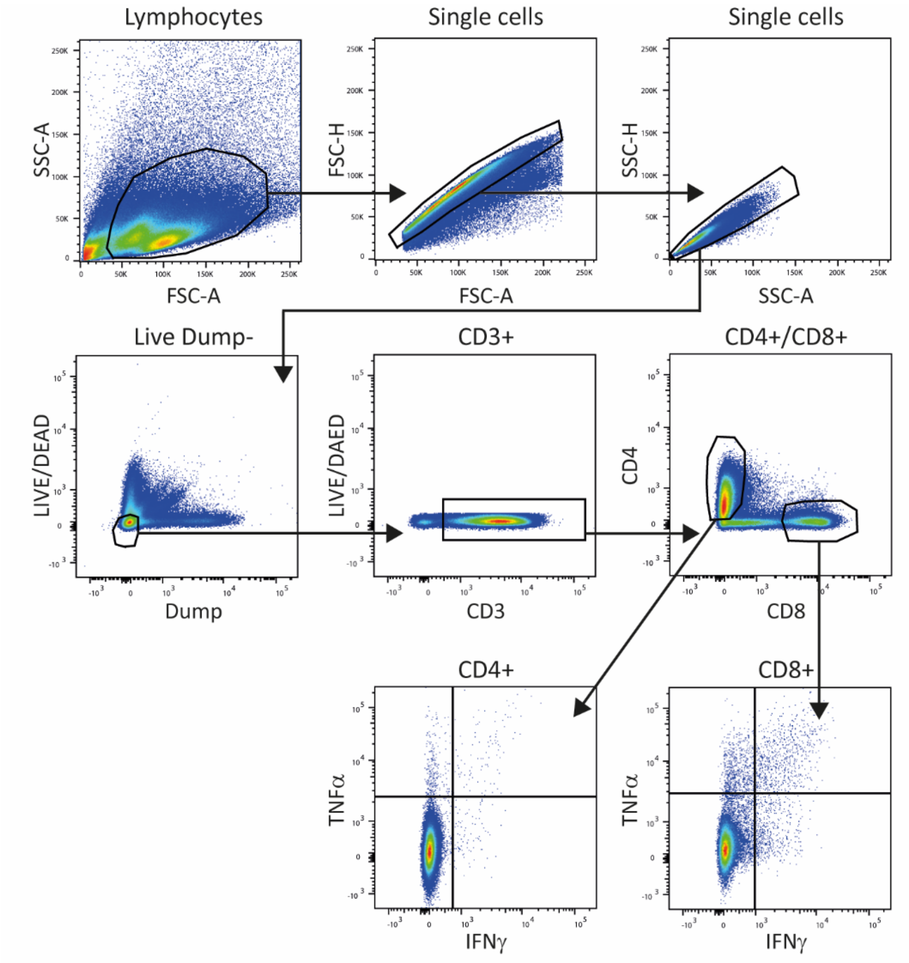

Supplementary figure 3. Gating strategy intracellular cytokine staining

First, the total lymphocyte population was gated followed by selection of single cells using both the forward and side scatter. Subsequently, the LIVE/DEAD and dump (CD14, CD16 and CD56) negative cells were selected to continue gating the CD3+ T cells. After gating on either the CD4+ or the CD8+ T cells IFNγ and TNFα positivity was determined using isotype control stains.

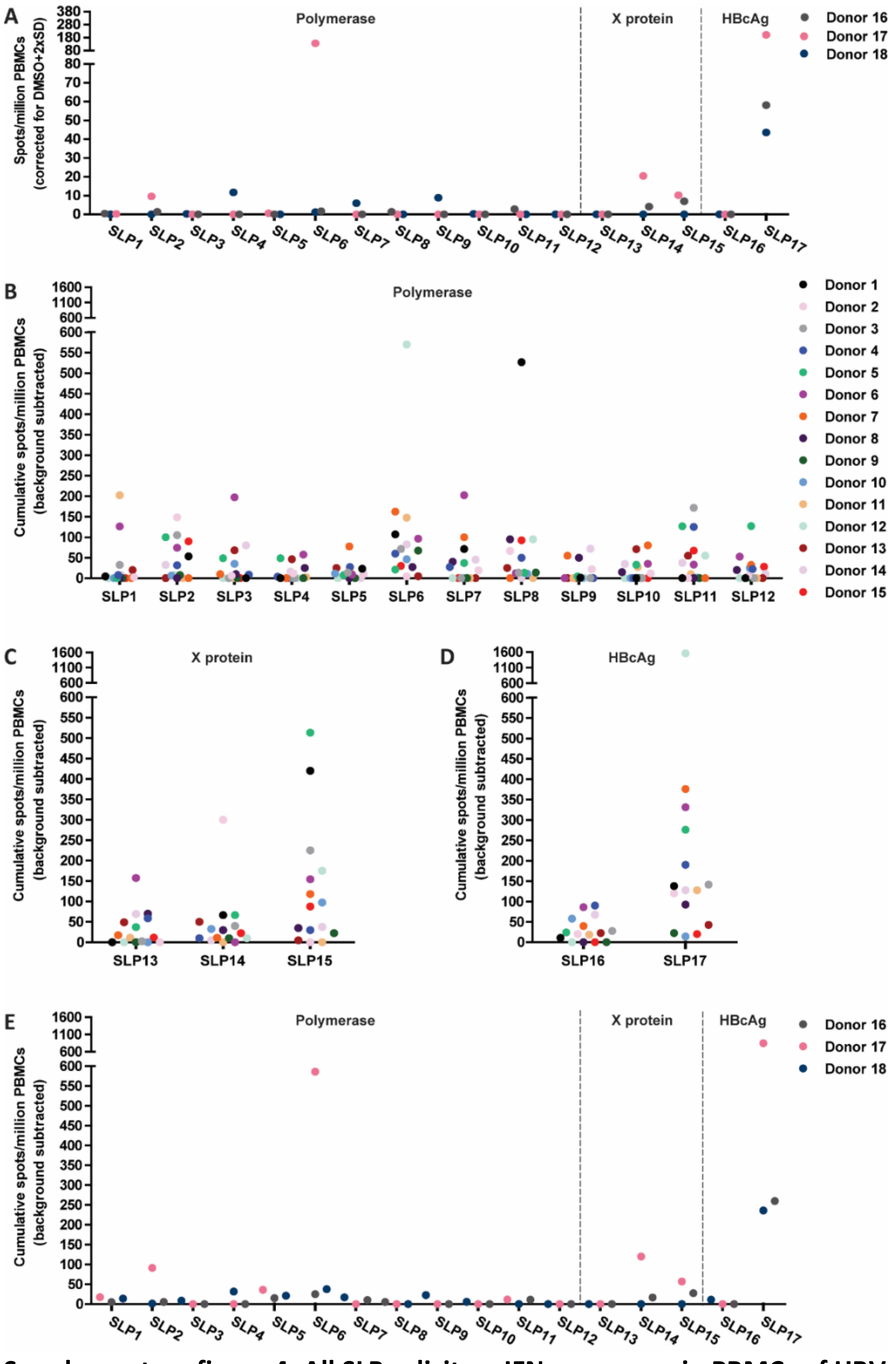

Supplementary figure 4. All SLPs elicit an IFNγ response in PBMCs of HBV resolvers

PBMCs of healthy blood donors that resolved an acute HBV infection somewhere in their past (rHBV) and of healthy controls were stimulated with the 17 SLPs to test their T cell activating capacity using an IFNγ ELISpot. A) Summary of ELISpot data (average spot count) of all 17 SLPs in PBMCs of healthy controls that have never been infected with HBV (n=3). B) Summary of ELISpot data for the polymerase SLPs. Spots are represented as the cumulative number of spots in four wells subtracted by the cumulative number of spots in the four DMSO control wells per million PBMCs. Each colored dot represents a different donor (n=15). C) Summary of ELISpot data for the X protein SLPs (n=15). D) Summary of ELISpot data for the HBcAg protein SLPs (n=15). E) Summary of ELISpot data (cumulative spots) of all 17 SLPs in PBMCs of healthy controls that have never been infected with HBV (n=3).

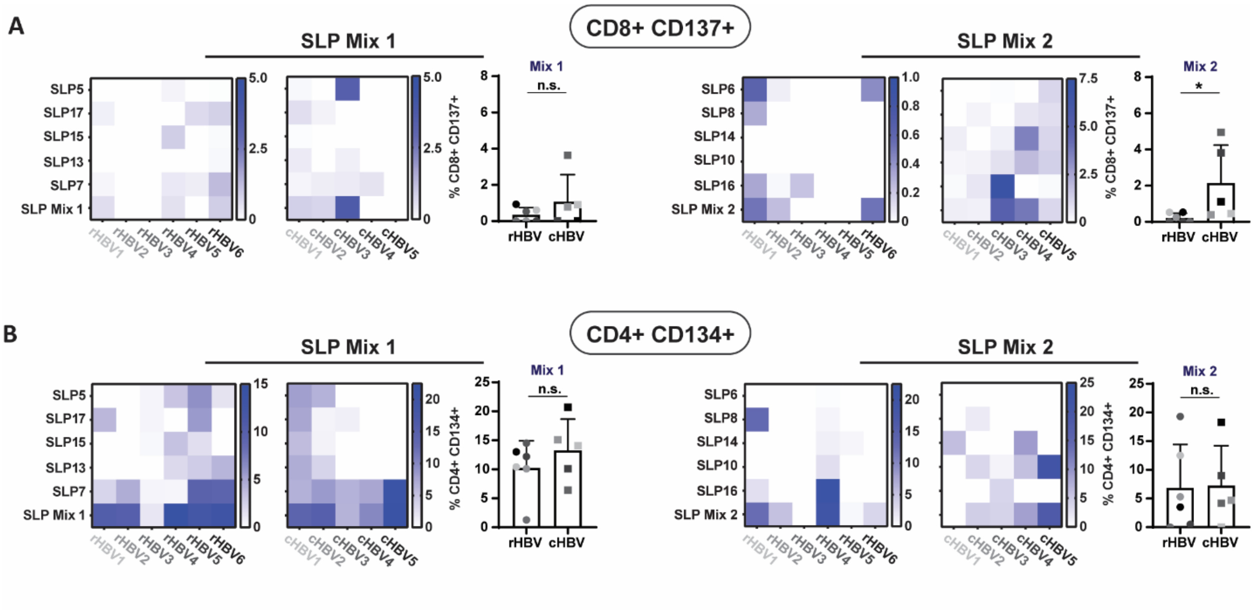

Supplementary figure 5. The best performing SLPs can induce activation of both CD8+ and CD4+ T cells
A) Heat maps were created for the expression of CD137 by CD8+ T cells in response to restimulation with the different single SLPs and the total SLP mix (left: expanded with SLP Mix 1, right: expanded with SLP Mix 2) for the rHBV (left) and cHBV patients (right). B) The expression of CD134 on CD4+ T cells after restimulation with the single SLPs or the SLP mix is displayed in the heat maps. Next to the heatmaps in both A and B is the summary of the response to the total SLP mix depicted including the number of responders out of the total number of tested individuals. An individual was marked responder when the response was positive after background correction. n.s. = non-significant, * $p<0.05$

SUPLEMENTARY TABLES

Supplementary Table 1

Donor #	HLA-A		HLA-B	
Donor 1	01:01 (A1)	02:01 (A2)	15:01 (B62)	38:01 (B27)
Donor 2	01:01 (A1)	02:01 (A2)	07:02 (B7)	44:02 (B44)
Donor 3 (rHBV6)	23:01 (A24)	11:01 (A3)	49:01 (unclas)	18:01 (B44)
Donor 4 (rHBV1)	03:01 (A3)	02:01 (A2)	07:02 (B7)	07:02 (B7)
Donor 5	01:01 (A1)	02:01 (A2)	08:01 (B8)	40:01 (B44)
Donor 6 (rHBV5)	01:01 (A1)	24:02 (A24)	08:01 (B8)	07:02 (B7)
Donor 7	68:01 (A3)	26:01 (A1)	51:01 (B7)	35:03 (B7)
Donor 8	01:01 (A1)	02:01 (A2)	15:01 (B62)	38:01 (B27)
Donor 9	26* (A1)	29* (A1 A24)	44:03 (B44)	07:02 (B7)
Donor 10	02:01 (A2)	25:01 (A1)	18:01 (B44)	07:02 (B7)
Donor 11	02:01 (A2)	25:01 (A1)	57:01 (B58)	40:01 (B44)
Donor 12	01:01 (A1)	02:01 (A2)	35* (B7)	37* (B44)
Donor 13	11:01 (A3)	03:01 (A3)	51:01 (B7)	41:01 (B44)
Donor 14 (rHBV4)	11:01 (A3)	33:01 (A3)	44:03 (B44)	35:01 (B7)
Donor 15	03:01 (A3)	11:01 (A3)	52:01 (B62)	51:01 (B7)
Donor 16	03:01 (A3)	11:01 (A3)	08:01 (B8)	07:02 (B7)
Donor 17	02:01 (A2)	24:02 (A24)	07:02 (B7)	07:02 (B7)
Donor 18	01:01 (A1)	24:02 (A24)	08:01 (B8)	07:02 (B7)
rHBV2	02:01 (A2)	02:01 (A2)	07:02 (B7)	40:01 (B44)
rHBV3	01:01 (A1)	03:01 (A3)	13:02 (unclas)	08:01 (B8)
cHBV1	11:01 (A3)	24:02 (A24)	n.d.	n.d.
cHBV2	33:01 (A3)	11:01 (A3)	n.d.	n.d.
cHBV3	02:01 (A2)	11:01 (A3)	14:01 (B27)	51:01 (B7)
cHBV4	03:01 (A3)	01:01 (A1)	50:01 (B44)	07:02 (B7)
cHBV5	02:01 (A2)	02:01 (A2)	13:01 (unclas)	51:01 (B7)

*HLA type is available in 2-digit resolution only.

Donor 3 and rHBV6, Donor 4 and rHBV1, Donor 6 and rHBV5, Donor 14 and rHBV4 are the same individual. Corresponding supertype is indicated between brackets. Unclas; unclassified is added when HLA type is not classified under a described supertype (28) n.d.; not determined. 4-digit HLA typing was performed using the Global Screening Assay (Illumina, executed by the Human Genomics Facility, Erasmus MC Rotterdam) and Axion HLA analysis software from Affymetrix.

Supplementary Table 2. Overview of the peptides tested in figure 5

PEPTIDE #	SLP #	PROTEIN	POSITION	HLA	SEQUENCE
**1**	7	Polymerase	500-508	I	KLHLYSHPI
**2**	7	Polymerase	502-510	I	HLYSHPIIL
**3**	7	Polymerase	503-511	I	LYSHPIILG
**4**	7	Polymerase	509-517	I	ILGFRKIPM
**5**	7	Polymerase	515-523	I	IPMGVGLSP
**6**	7	Polymerase	499-514	II	RKLHLYSHPIILGFRK
**7**	7	Polymerase	508-523	II	IILGFRKIPMGVGLSP
**10**	14	X protein	58-66	I	LPVCAFSSA
**11**	14	X protein	62-73	I	AFSSAGPCALRF
**12**	14	X protein	67-75	I	GPCALRFTS
**13**	14	X protein	52-66	II	HLSLRGLPVCAFSSA
**14**	14	X protein	58-72	II	LPVCAFSSAGPCALR
**16**	16	Core	8-16	I	EFGATVELL
**17**	16	Core	18-26	I	FLPSDFFPS
**18**	16	Core	18-27	I&II	FLPSDFFPSV
**20**	16	Core	23-31	I	LPSDFFPSV
**21**	16	Core	3-19	II	IDPYKEFGATVELLSFL
**22**	16	Core	12-27	II	TVELLSFLPSDFFPSV
**23**	16	Core	23-37	II	FFPSVRDLLDTASAL
